# Controlled inoculation provides insight into western redcedar resistance to multiple root- and butt-rot pathogens

**DOI:** 10.3389/fpls.2025.1669570

**Published:** 2025-10-14

**Authors:** Jun-Jun Liu, Sydney Houston, Mike Cruickshank, Arezoo Zamany, Isabel Leal, Cosmin Filipescu

**Affiliations:** ^1^ Pacific Forestry Centre, Canadian Forest Service, Natural Resources Canada, Victoria, BC, Canada; ^2^ Department of Biology, University of Victoria, Victoria, BC, Canada

**Keywords:** artificial inoculation, latent infection, molecular diagnosis, root- and butt-rot diseases, western redcedar

## Abstract

Western redcedar (*Thuja plicata* Donn) is one of the most valuable forest species in western North America, but high incidence of root- and butt-rot diseases has resulted in large economic losses. During a long history of host–microbe co-evolution, redcedar has gained highly quantitative disease resistance to these pathogens compared to other conifer species. Despite this, genetic disease resistance has rarely been incorporated into redcedar breeding programs. This study evaluated redcedar resistance to the root- and butt-rot diseases caused by eight wood decay fungi. Two artificial inoculation methods, using wood block-stick and dowel-plug inoculums, were developed for infection of three-year-old seedlings under controlled greenhouse conditions. Disease symptoms and infection processes were assessed over 18 months post inoculation. Disease incidence rates ranged from 10% to 60% for five pathogens (*Armillaria ostoyae, Coniferiporia weirii*, *Heterobasidion occidentale*, *Poriella subacida*, and *Postia balsamea*). Among these, only *C. weirii* and *Poriella subacida* caused symptoms of both wood discoloration and decay. Infection processes varied among the five pathogens. The remaining three decay fungi (*Porodaedalea pini*, *Postia sericeomollis*, and *Obba rivulosa*) did not cause obvious disease symptoms. However, molecular diagnosis using next-generation sequencing of the internal transcribed spacer region (ITS-NGS) detected target pathogens in asymptomatic but inoculated seedlings. These latent infections were characterized by high incidence rates and intermediate levels of molecular infection severity (MIS), which significantly impaired seedling growth. The continuous MIS variation among asymptomatic seedlings highlights latent infection as a key quantitative trait for screening resistance in western redcedar. This study provides essential insights into disease development and latent infection in western redcedar, contributing to improved prediction of disease outbreaks, forest health management, and the development of early intervention strategies. The inoculation methods and molecular diagnostics established here offer valuable tools for integrating disease resistance into western redcedar breeding programs.

## Introduction

Western redcedar (*Thuja plicata* Donn ex D. Don) is an important forest species across the Pacific Northwest of North America with large ecological, cultural, and economic significance (Antos et al., 2016). British Columbia (BC), Canada, has the world’s largest standing redcedar stocks representing over $1 billion in economic activity annually in the BC forest industry (Gregory et al., 2018). Although this conifer species has shown high level of resistance to root- and butt-rot diseases compared to other conifers, frequent disease incidence has been reported in BC redcedar stands, suggesting a potential outbreak or endemic presence requiring further investigation. As redcedar stands mature, disease incidence rates increase from ~18% by age 50 years to 35% by age 100 of BC coastal stands, 80% by age 100 of BC interior stands, and close to 100% by age 300–400 years of both BC coastal and interior stands (Buckland, 1946), as well as in Southwest Alaska (Kimmey, 1956). More recent surveys have reported similar disease incidence rates in BC interior sites (Morrison et al., 2001; Cruickshank et al., 2018). These increasing wood-decay rates over time indicate the possibility of latent infection of decay-causing pathogens in asymptomatic redcedar trees.

High incidence of root- and butt-rot diseases and extensive damage have resulted in large economic losses for the forest industry (Sturrock et al., 2017; Bogdanski et al., 2023). Wood decay-caused loss accounted for up to 30% of total gross wood volume for redcedar (BC Forest Service, 1957; USDA, 2012), which is higher than the 12% average decay loss of other conifer species (BC Forest Service, 1957). Although the wood decay does not kill the trees outright, it decays the lower portion of the bole, causing stem breakage by winds. However, the greater concern is economic. In addition to merchantable volume loss, decay also increases harvesting costs through stem breakage after falling or skidding (Renzie and Han, 2001; Sturrock et al., 2017).

Redcedar root- and butt-rot diseases are caused by several soil fungal pathogens (Sturrock et al., 2017), mainly including four white rot fungi: *Coniferiporia weirii* (Murrill, Conw), *Obba rivulosa* (Obbr), *Poriella subacida* (Pors), and *Porodaedalea pini* (Brot. Murrill, Porp); and two brown rot fungi: *Postia balsamea* (Murrill, Posb) and *Postia sericeomollis* (Poss). *Armillaria ostoyae* (Armo) and *Heterobasidion occidentale* (Heto) also infect and cause root-rot diseases in living redcedar trees (Otrosina and Garbelotto, 2010; Sturrock et al., 2017). However, both fungi are generally more aggressive and virulent in other conifer hosts such as Douglas-fir, true firs, and others (Cleary et al., 2012; 2021; Liu et al., 2022). To date, little information is available for their pathogenicity and infection process in redcedar.

Conw, previously named as *Phellinus weirii* or *Poria weirii* (Zhou et al., 2016), is considered as the most important white rot fungus affecting redcedar (Hagle, 2006). Its native North American hosts include redcedar, yellow-cedar (*Callitropsis nootkatensis* D. Don), and incense-cedar (*Calocedrus decurrens*) (Wang et al., 2022). As the major fungal pathogen on living redcedar trees, it has caused the most serious wood decay in most old-growth interior redcedar stands (Sturrock et al., 2017). Conw-caused wood decay usually extended 2–3 m up the boles with the most extreme cases reaching 10 m in living redcedar trees. Because of decay-related losses (e.g., butt rot, heart rot, and pocket rot), only 32% to 49% of total harvested volume was usable in BC Interior Cedar-Hemlock forests aged 300–350 years (Renzie and Han, 2001).

Pors (previously named as *Perenniporia subacida*, Chen et al., 2021) causes white rot in a wide range of conifer species, as well as in hardwoods in temperate and tropical forests, rating as one of four important white rot decay fungi in redcedar, especially in BC coastal redcedar stands (Buckland, 1946). Like Conw and Heto, Pors does not produce rhizomorphs in soil, its mycelium spreads through root contacts to neighboring trees, causing butt rot, leading to structural weakening and decay in the lower trunk and roots (Tabata et al., 2009). Porp is widespread in BC and across North America, especially in older conifer stands. Redcedar is one of its primary conifer hosts, causing white pocket rot in the heartwood of living trees (Sturrock et al., 2017). Obbr was not generally considered a pathogen in living trees, but well-known for its ability to selectively degrade lignin in dead wood, particularly in conifers (Miettinen and Rajchenberg, 2012). However, this fungus was isolated from decaying redcedar wood (Lim et al., 2005) and has been the most common fungus observed in the coastal redcedar stands (Sturrock et al., 2017). Posb (previously named as *Tyromyces balsameus* Peck) is a brown cubical rot in living and dead conifers (Rizzo and Harrington, 1988). Poss is a pathogenic fungus, causing brown cubical butt and pocket rot and affecting the heartwood of living conifers. Poss was ranked second only to Conw in amount of decay volumes in living redcedar trees in the interior of the western USA (Hagle, 2006). Although these mentioned fungi have been detected in redcedar and considered as pathogens of living redcedar (Sturrock et al., 2010), it still awaits controlled inoculation to fully understand the infection processes for their invasion into living redcedar tissues for development of decay diseases.

Management of root- and butt-rot diseases remains challenging due to lack of information, especially related to both fungal pathogenicity and host resistance or tolerance to pathogens. Redcedar has strong durability to decay pathogens, which was associated with secondary metabolites of its heartwood (Morris and Stirling, 2012). To date, few investigations have been performed for infection and decay disease development in redcedar (Sturrock and Reynolds, 1998; Cleary et al., 2012) and related conifer species (Liu et al., 2022). Earlier breeding program objectives primarily focused on tree growth and adaptability as well as heartwood durability (Russell and Yanchuk, 2012). In recent years, increasingly frequent and severe summer droughts have likely heightened the vulnerability of western redcedar to canopy dieback and mortality (Andrus et al., 2024). To address these challenges, current breeding programs have begun screening redcedar populations for enhanced resistance to both fungal pathogens and drought stress (Gamal El-Dien et al., 2022; Aldana et al., 2024).

As an initial effort for redcedar disease resistance breeding, this study aimed to evaluate host genetic resistance to different wood decay pathogens through development of controlled inoculation methods. Artificial inoculums of wood block-stick and dowel-plug were compared for their effectiveness to infect young seedlings under controlled greenhouse conditions. The fungal infection process was monitored through dissecting living tissues and molecular diagnosis, and resistance of young seedlings to different decay fungal pathogens was evaluated by assessment of disease symptoms. The results of this study provide foundational information for developing genetically resistant redcedar varieties, which can significantly enhance the sustainability and productivity of the redcedar industry.

## Materials and methods

### Plant materials

Two full-sib seed families of coastal western redcedar (*Thuja plicata* Donn) were used in this study (**Supplementary Table S1**): #8422 (#584 x #658) and #8423 (#658 x #786). These two seed families were half-sibs with one shared parent tree (#658). They were provided by the Cowichan Lake Research Station (Cowichan Lake, BC).

### Fungal inoculation procedures

Eight wood decay fungal species were used for greenhouse inoculation tests (**Supplementary Table S2**), including: *Armillaria ostoyae* (Armo), *Coniferiporia weirii* (Conw), *Heterobasidion occidentale* (Heto), *Poriella subacida* (Pors), *Postia balsamea (*Posb), *Porodaedalea pini* (Porp), *Postia sericeomollis* (Poss), and *Obba rivulosa* (Obbr). Fungi of interest were cultured on 3% malt extract agar media in petri dishes.

**Figure 1:**
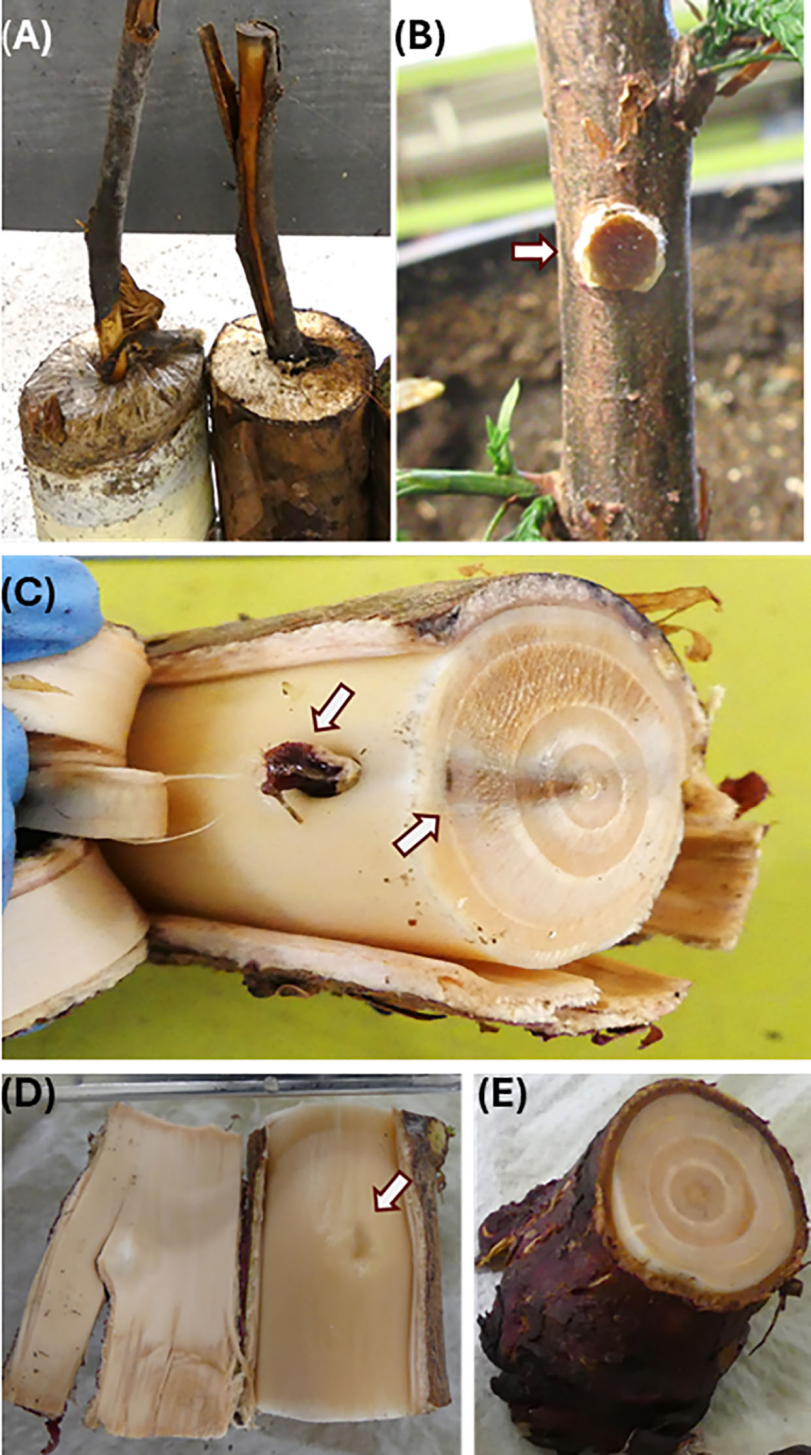
Inoculation symptom assessment of redcedar seedlings using controlled inoculation methods. Assessment of disease symptom was performed at 18 months post inoculation. **(A)** Block-stick inoculum from pot soils at 18 months post inoculation. **(B)** Dowel-plug inoculum just inserted at the stem wounding site. **(C)** Disease symptom of wood discoloration caused by *Postia balsamea* dowel-plug inoculum. One arrow points at the callus surrounding the inoculation site for the dowel-plug inoculum, and another arrow points at black discoloration zone in the stem cross section. **(D)** Stem wounding site healed with formation of callus in a control seedling. **(E)** Stem cross section of control seedling.

Two methods, termed as the block-stick and dowel-plug methods, were used for inoculation of seedlings (**Supplementary Table S1**). Inoculum units for the block-stick inoculation method were prepared following a previous protocol (Sturrock and Reynolds, 1998; Cruickshank et al., 2020) with modification. In brief, for the block-stick inoculum units (**Figure 1A**), stems of juvenile red alder (*Alnus rubra* Bong.) were harvested and cut into wood block segments with ~ 5 cm diameter x ~ 6 cm length. A hole of 9.5 mm diameter was drilled longitudinally 3–4 cm through the center of each stem segment. Twenty to 25 wood blocks were then placed side by side into an autoclavable bin. Distilled water was added to each bin so that the top of the wood blocks was just covered with water. The bin was then autoclaved for 75 min at 121°C. After autoclaving, excess water was drained, and 8 wood blocks were placed in two stacks of 4 with the drilled hole side pointed up. Both stacks of wood blocks were placed in a 20 x 56 cm autoclavable mushroom spawn bag with 0.2-micron microporous patch to allow gas exchange. The tops of bags were left open and then autoclaved for 75 minutes at 121°C slow exhaust cycle. After autoclaving, the tops of bags were folded over and held with 1 inch fold back clips, and then cooled in a sterile flow hood overnight. Wood bags were inoculated aseptically by placing 2 cm square cubes from half of a fungal colonized petri plate per bag, distributed evenly on the edge of each wood block. The inoculated bags were re-clipped and placed in the dark at 10°C in a walk-in cooler to allow the fungus to grow for up to one year.

The block-stick transfer sticks were prepared using two-year-old branches of various species (western redcedar, western hemlock, and Garry oak). Based on the preferred host species of each pathogen, wood sticks from different host trees were used in the block-stick inoculations. These branches were harvested and cut into sticks with diameter at one end slightly larger than the block hole. They were rinsed lightly with tap water and allowed to air dry. Once the alder wood blocks were colonized, a redcedar branch stick was inserted into each hole of the blocks inoculated with Conw and Pors; Garry oak sticks were used for blocks inoculated with Armo; hemlock branch sticks were used for blocks inoculated with Heto. Sticks were inserted by trimming one end, so it fit tightly into the block hole and the bark had firm contact with the block around the hole. The branch sticks were trimmed 1” shorter than the depth of the pot. The block end was covered with a plastic bag, taped closed, and aluminum foil was placed over the block to keep it dark. After removal of the PVC tube from the seedling pots, the stick of the unit was inserted into the hole left behind by the PVC tube in each pot, with the block remaining above ground. Excess space around the branch stick was filled with soil. For the control group in the block-stick inoculation method, a PVC tube was inserted into the potting soil to simulate the physical disturbance without introducing fungal inoculum.

For the dowel-plug inoculation method, inoculum units were prepared using wood dowels of stem cross sections of 8 mm, and they were obtained from a 35-year-old western red cedar tree. Dowel plugs at 5 mm diameter x 3 mm thickness were prepared from the heartwood using an increment borer. The dowels were autoclaved in bags for 30 min, cooled in a laminar flow hood, and placed on colonized petri dishes until the wood dowels were colonized. An increment borer was used to remove a bark/stem section from each of the greenhouse trees between internodes at approximately 10 cm from the soil. A colonized dowel-plug was inserted and trimmed to fit flush with outer bark (**Figure 1B**). The inoculation point was wrapped with parafilm around the stem and tied with grafting rubber to hold the plug-in place. For the control group in the dowel-plug inoculation method, a sham-wound control was implemented by inserting an uncolonized plug into the stem wound site.

One-year-old seedlings were planted in one-gallon pots in the spring of 2016. A 1” PVC tube was inserted offset from center and vertically into the container to accommodate the stick from the inoculation unit. Seedlings were moved into two-gallon pots in the spring of 2017. Inoculation was performed during November 7^th^ to 9^th^, 2017 on three-year-old seedlings. A total of 88 seedlings were used for inoculation trials with 10 seedlings per pathogen, and 8 seedlings as controls (**Supplementary Table S1**). Seedlings were potted individually and placed in a randomized complete block design within the greenhouse to minimize positional effects.

A peat-vermiculite-sand (1:1:1) mix was used as pot soils, and no additional fertilizer was added. In all cases the soil in the pots was kept moist but not wet with ambient humidity. The seedlings were placed on a bench in a temperature-controlled glass greenhouse kept between 12-18°C controlled by forced air and evaporative coolers. Seedlings were automatically watered with drippers. No supplemental electric lighting was provided, relying solely on natural sunlight. No additional pests were observed during the inoculation trials, and therefore no further pest management interventions were required.

In many cases, the fungus could be seen moving across the top of the soil from the wood block and sometimes a short way up the stem of the tree. The fungal movement on the soil occurred in the fall when the days became shorter. It was important to keep the soil surface from completely drying out at this time. Greenhouse conditions were regularly inspected to minimize the risk of cross-contamination between pots.

### Symptom assessment of fungal infection

Disease symptoms were assessed, and redcedar tissues were sampled at 18 months post inoculation during June 25^th^-July 3rd, 2019. Seedling heights and stem diameters at ground level were measured. Stem (including bark) diameter was measured for each seedling at the soil line using a Vernier caliper at two positions 90° apart. Inoculum units used for the block-stick method were checked visually for fungal presence in the wood blocks and sticks. Inoculum units were considered successful if ectotrophic mycelium had grown at least half-way down the branch sticks and transfer of fungal pathogens to the seedlings was confirmed by presence of ectotrophic mycelium on roots. The mycelium on the roots and stick was distinct and easily seen visually.

For destructive evaluation of disease development, stems of seedlings inoculated by the dowel-plug method were cross-sectioned at the inoculation sites. For the seedlings inoculated by the wood block-stick method, stem cross sections were checked at the root collar and the lower part of the stem at the soil line where the primary lateral roots arise from the root collar. Stem cross-sections were checked for the presence or absence of dark staining and wood decay inside the stem tissues as an indicator of xylem decay caused by root rot pathogens (Sturrock and Reynolds, 1998).

To detect decay fungi in redcedar tissues by molecular diagnosis, root collar wood and fine root samples were taken from all seedlings at the end of inoculation trials. Tissue samples were cleaned by rinsing with tap water to remove residual soil and debris; Following surface sterilization with 70% ethanol, a subset of tissue samples was cultured on petri dishes to confirm colonization by the inoculated fungi.

### Molecular diagnosis to detect and quantify latent infection in seedlings

Genomic DNA was extracted from redcedar fine roots using a DNeasy Plant Pro Kit (Qiagen), and from root collar wood tissues using a hexadecyltrimethylammonium bromide (CTAB)-based protocol (Healey et al., 2014). Next-generation sequencing of internal transcribed spacer regions (ITS-NGS) was performed to detect and quantify the target fungus in total DNA extracted from redcedar living tissues (Houston et al., 2025). The full-length ITS region was initially amplified using polymerase chain reaction (PCR) with fungal-specific primers ITS1F and ITS4 (White et al., 1990; Gardes and Bruns, 1993). Subsequently, the amplicon libraries of the ITS1 regions were prepared using primers ITS5 and ITS2 (White et al., 1990) and sequenced on the Illumina MiSeq platform following the standard protocol (Schirmer et al., 2015; Illumina, 2018). MiSeq libraries were prepared with sample-specific tags and pooled in equimolar concentrations across all samples, resulting in an average sequencing yield of 0.2 million of 250-bp paired-end (PE) reads per sample. PCR without genomic DNA template was included as negative control for assessment of potential contamination.

FASTQ files of MiSeq 250-bp PE reads were first filtered to remove the reads with low quality scores using CLC Genomics Workbench (ver 23.0, Qiagen). The clean reads were clustered and annotated using the Operational Taxonomic Unit (OTU) Clustering (ver 2.6) in the CLC Microbial Genomics Module (ver 23.0). Unique reads of each sample were assigned an OTU at 97% sequence similarity using the UNITE-reference database (https://unite.ut.ee/#main; Sh_general_release_seq161,355; 16.10.2022).

Relative abundance of infected fungal pathogens (Ps) was calculated as the target read counts in proportion to the total fungal reads for each redcedar sample. The incidence of latent infection was estimated by presence of pathogens in living tissues of asymptomatic seedling inoculated by each fungal pathogen at threshold of Ps = 5 x 10^-5^. Molecular infection severity (MIS) was calculated for each target pathogen and each redcedar seedling with the following formula:


(1) $$\text{MIS}=6+{\text{log}}_{10}(\text{Ps}+10^{-6})$$

where Ps values were transformed by logarithm and with MIS ranging from 0 to 6. The linear regression model was tested and visualized using a scatter plot to evaluate the impact of MIS on seedling growth, including the 95% confidence interval.

### Statistical analyses

To test for the effect of fungal type and inoculation method on the presence of decay/discolor or the presence of fungal ITS, a binary generalized linear mixed-effects model (GLMM) was first attempted according to:


(2) $$\text{Prob}\;(ITS_{ijkm})=\text{p}=1\;/\;exp(\;FungalID_{ij}+Inoc_{ik}+Family_{im})\;$$

where ITS is the binary presence or absence of ITS (0=absent, 1= present) in tree i at sampling age for fungal type j, inoculation type k and family m. Fungal ID are the categorical fixed effect of the eight types of fungi with control as the reference level. Inoc is the categorical fixed effect of inoculation method dowel-plug or block-stick. Family is the categorical random effect of half-sibling family. Interactions were only included according to a significant (p ≤ 0.05) likelihood ratio test, but none were significant. The model for decay/discolor presence was the same except the response was $Decay_{ijkm}$ (0 = absent, 1 = present).

Both models indicated that variance components for the random effect were near zero and that the model was overfit. The random effect of family was removed, and a generalized linear model (GLM) with a binary response identical to Equation 2 but without the random effect was used. For the ITS response model, significant effects of the FungalID were found. Odds ratios relative to the control were determined for each fungus. Odds ratios greater than one indicate an increase in the odds of ITS presence relative to the control, and ratios less than one indicate the decrease in odds of ITS presence relative to the control, or relative to the block-stick inoculation method for Inoc. For the decay response model, none of the terms in Equation 2 were significant (p > 0.6) and this was not considered further. Models were checked for goodness of fit according to a Hosmer–Leme show goodness-of-fit test which showed adequate fit for both responses (p ≥ 0.36).

To test for differences in height and diameter due to infection, a liner mixed effect model (LME) was used according to:


(3) $$Height_{ijkm}=FungalID_{ij}+Inoc_{ik}+Family_{im}+\epsilon_{ijkm}$$

where ‘Height’ is the height of the tree i at sampling age in cm for fungal type j, inoculation method k and family m; FungalID, Inoc, and Family are as described in Equation 2; ϵ is the residual error. A compound symmetry covariance matrix (corCompSymm) was accommodated in the model to account for correlation of trees within families. Interactions were only included according to a significant (p ≤ 0.05) likelihood ratio test, but none were significant. The model for diameter of trees by fungus and inoculation method was identical to Equation 3 except that the response was Diameter_ijkm in cm. Family had a minor effect on both model explaining only 3% or less of the error term, but Family was retained to show that it was accounted for. LME models were checked for normality and homoscedasticity by q-q plots and plots of residuals against fitted values, respectively.

To test for the effect of ITS or decay presence on tree height and diameter, we tested a model similar to Equation 3. The term for Inoc in Equation 3 was dropped and replaced with ITS according to:


(4) $$Height_{ijkm}=FungalID_{ij}+ITS_{ik}+Family_{im}+\epsilon_{ijkm}$$

where the terms were identical to Equation 3, including the covariance matrix except that ITS was the categorical effect of fungal ITS k in tree i (0 = absent, 1 = present). The model for Diameter was similar except that the response was Diameter_ijkm. A similar set of models was tested for height and diameter using decay presence and absence in place of ITS, but neither model was significant. The only significant effect was for the effect of ITS on height of the trees. Similarly, Family had minor effects on all models as stated above, and was only included for accountability.

## Results

### Incidence of disease symptoms of wood-decay and discolor

No morphological change (such as needle discoloration, or loss of foliage) was observed in the inoculated seedlings as compared to non-inoculated controls. Wood discoloration and decay were the most visual disease symptoms in the inoculation trials of this study. Disease symptoms were observed in seedlings inoculated by five fungal species, calculating the disease incidence rates at 10%, 20%, 40%, 50%, and 60% for Posb, Armo, Conw, Heto, and Pors, respectively. In contrast, no disease symptoms were observed in seedlings inoculated by Porp, Poss, and Obbr, or the non-inoculated control seedlings.

Block-stick and dowel-plug inoculum (**Figures 1A, B**) were compared for successful disease developments caused by the decay pathogens. The block-stick inoculum caused disease incidence rates higher than the dowel-plug for Armo (33% vs. 0%) while the dowel-plug caused higher disease incidence rates than the block-stick inoculum for Conw (75% vs. 17%) and Posb (25% vs. 0%). The block-stick and dowel-plug inoculums caused similar disease incidence rates for Heto (50% vs. 50%) and Pors (67% vs. 50%). In total of these five pathogens that caused disease symptoms in this study, block-stick and dowel-plug inoculums caused disease incidence rates of 33% and 40%, respectively, without significant difference between them (X^2^ test, p = 0.23).

By combining data from the two inoculation methods and five symptom-causing decay pathogens, the families #8422 and #8423 had a disease incidence rate at 28% and 44%, respectively, but without significant difference between families (X^2^ test, p = 0.24). In consistency, the GLMM test further confirmed that the presence of decay/discolor (Equation 2) was not affected by fungal type or inoculation method (p > 0.60). As expected, variance components for the random effect of family type were near zero, likely due to the two families being half-sibs.

### Disease development processes

Of five symptom-causing pathogens, disease development showed two different patterns. Conw, Pors, and Posb showed similar infection processes, characterized by radial spread from the inoculation or infection sites inward to the stem center. Posb successfully infected seedlings only from dowel-plug inoculum and callus was formed in the cambium tissue surrounding the inoculation site (**Figure 1C**). Its infection caused wood discoloration in the wood tissues of the 1^st^ to 3^rd^ years of seedling growth, but it appeared that inoculation did not affect stem wood tissues grown after the inoculation year, the 3^rd^ and 4^th^ year of seedling growth (**Figure 1C**). A black color zone was formed in the wood tissues of the 3^rd^ year late summer/fall growth, preventing fungus to spread outward to wood tissues that were developed during the 4^th^ and 5^th^ year of seedling growth. Despite discoloration in the first three rings of seedling growth, no decay had developed at 18-mpi. In contrast, the wound for dowel-plug insertion was healed by callogenesis in the non-inoculated control seedlings (**Figures 1D, E**).

**Figure 2:**
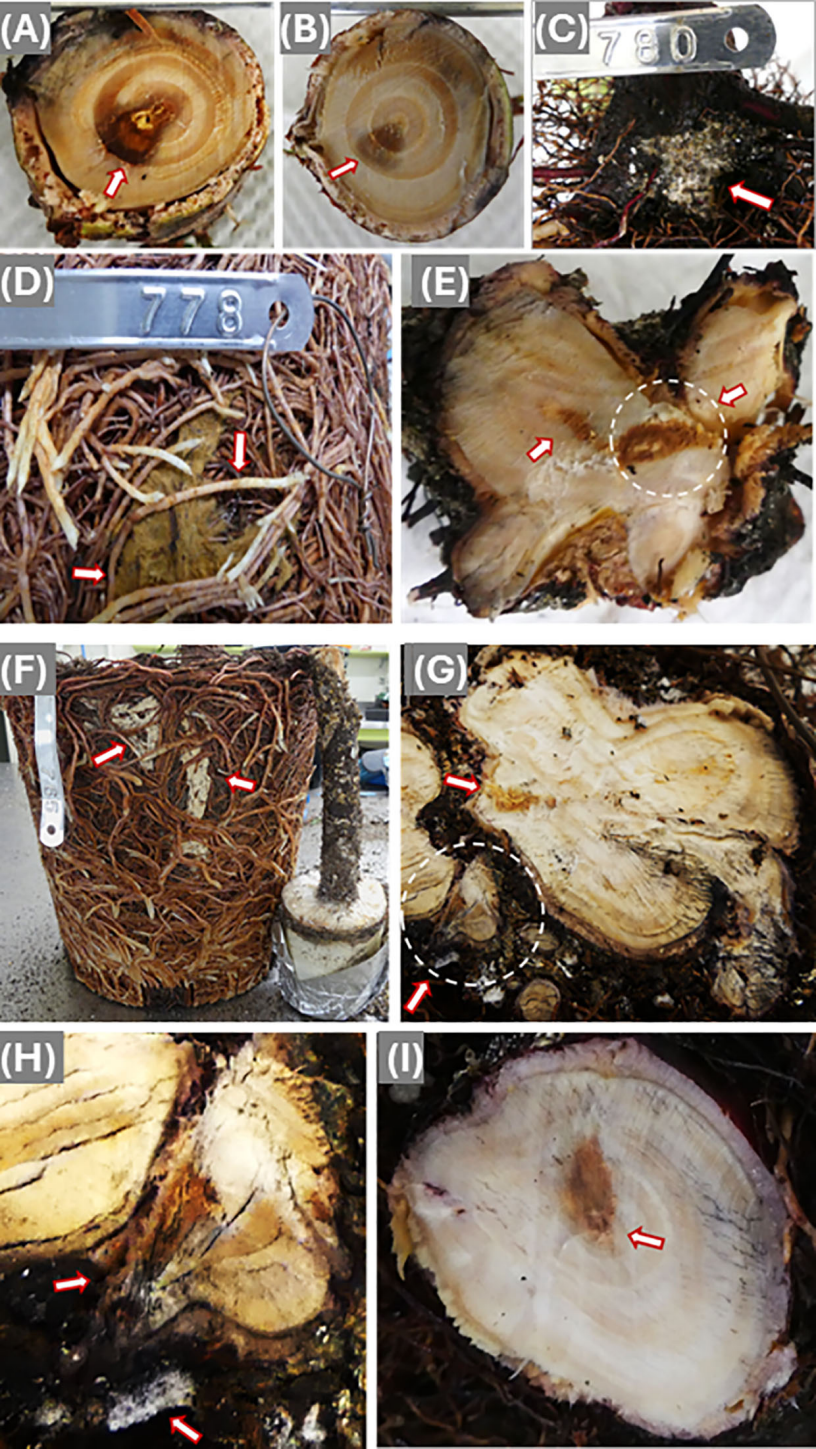
Disease symptoms caused by infection of *Coniferiporia weirii* **(A–E)** and *Poriella subacida* **(F–I)**. **(A)** Bottom side cross section of a stem segment, which was cut immediately above the dowel-plug inoculation site. Arrow points at black discoloration zone. Wood decay appeared at the wood center. **(B)** Topside cross section of the same stem segment of **(A)**, wood discoloration became lighter with smaller area in wood tissues of the 1^st^ and 2^nd^ year of seedling growth. **(C)** Mycelium from block-stick inoculum colonized the bark of root collar and fine roots directly **(D)** Mycelium colonized the surface of the root system, and arrows point at current year fine roots infected by *C*. *weirii*. **(E)** Cross section of infected root collar. The arrow-pointed circle shows a wood area with infection from one side of lateral root, but decay appeared at the wood center. Another arrow points at discolored area in the root collar wood tissues of the 1^st^ and 2^nd^ year of the seedling growth. **(F)** The block-stick method showing a root well colonized by *P. subacida* mycelium. **(G)** Cross sections of lateral primary roots, showing infection starting from bark and spreading inward to wood tissues. The circled area was magnified in **(H)**. **(H)** Colonization of mycelium on the bark of one lateral primary root as pointed by one arrow. Another arrow points at a rotten secondary root from which the fungus spread to the primary root and decayed one side of the root. **(I)** Cross section of root collar at a higher position, showing discoloration in the wood tissues of the 1^st^ and 2^nd^ year seedling growth, and decay appeared in the stem pith.

A similar pattern was observed for Conw dowel-plug inoculum, but the inoculum killed wood tissues during the inoculation year (the 3^rd^ year of seedling growth) at the inoculation site and a black color zone was formed in the wood tissues of the 2nd year late summer/fall growth. Wood decay was also observed in the tissues of the 1^st^ and 2^nd^ year of seedling growth (**Figure 2A**). Infection-caused discoloration at the inoculation sites moved vertically in the stem of the 3rd year seedling growth (**Figure 2B**).

Mycelium growth from the Conw block-stick inoculum was observed on the surface of root systems, including the youngest fine roots directly grown out from root collar (**Figure 2C**) and the fine roots of current year growth (**Figure 2D**). In the cross-section of one of the main lateral roots, wood discoloration started from cambium tissues at the infection site, and extend inward and caused decay in the pith (**Figure 2E**). At the root collar section, wood discoloration and decay were restricted to the wood tissues of 1^st^ and 2^nd^ year seedling growth, without symptoms inside wood tissues of the 3^rd^ and 4^th^ year seedling growth (**Figure 2E**). This disease development pattern was similar to that observed from infection by dowel-plug inoculum but typically involved more than one penetration area caused by mycelium on different lateral roots.

A similar infection process to that described for Conw was observed in several seedlings infected by Pors. Following inoculation using stick of the block inoculum, the root system was extensively colonized by the mycelium (**Figure 2F**). Wood discoloration started from infection sites of several primary lateral roots. Extensive wood decay was sometimes observed in the junction between the primary and secondary lateral roots when the secondary root was killed, which allowed the pathogen to then infect the larger root (**Figures 2G, H**). Cross sections showed that wood decay spread from one or more main lateral roots to the root collar pith (**Figure 2I**).

**Figure 3:**
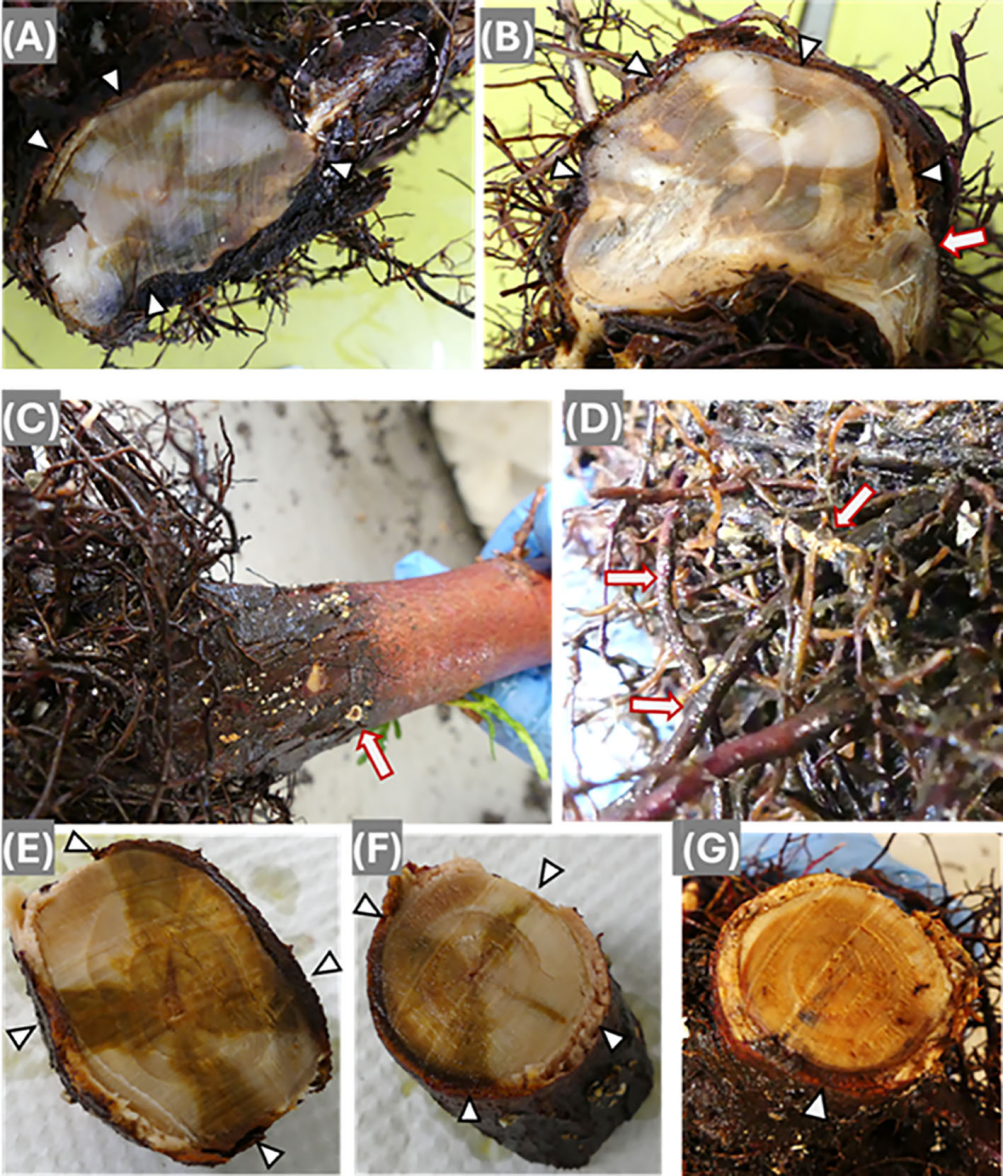
Disease symptoms following infection by *Armillaria ostoyae* **(A, B)** and *Heterobasidion occidentale* **(C–G)**. **(A)** Cross section of root collar. Triangle-like arrows point at discolored wood sites starting from cambium with spread inward to stem wood center. The circled area shows mycelium colonization beneath the bark of one lateral primary root. **(B)** Cross-section of root collar. Triangle-like arrows point at discolored wood area and a regular arrow point at discoloration in the wood center of one primary lateral root. **(C)** Bark surface of a root collar. *H*. *occidentale* mycelium colonized as white-colored spots, and the arrow points at one fruiting body at early developmental stage. **(D)** Mycelium colonized on the secondary roots. **(E)** Bottom side section of a root collar segment with larger sizes of discolored areas. **(F)** Topside section of the same root collar segment of **(E)** with smaller sizes of lighter discolored areas. Arrows point at discolored area from cambium with inward spread. **(G)** Cross section of root collar. Arrow points at the darker discoloration zone in the cambium of the infection side, and lighter discoloration spread over almost the whole area of the wood section.

Armo and Heto showed a different infection process with radial spread from the epidermis. *Armo mycelia* were well colonized beneath the main root bark (**Figure 3A**). At root collar sections, discoloration spread from several locations on the outer wood surface to the stem center, but no decay was observed in the discolored wood area (**Figure 3B**).

For seedlings inoculated by Heto, fruiting bodies were observed during the early development stage on root collar bark (**Figure 3C**), and the secondary roots were well colonized by the fungal mycelium (**Figure 3D**). Similarly to infection by Armo, discoloration spread from the wood surface to the stem center from one or several locations (**Figures 3E, F**). In the most serious case, the discoloration almost covered the entire wood area of the root collar section (**Figure 3G**). Like infection by Posb, wood decay had not yet developed in the seedlings infected by Armo and Heto, indicating redcedar resistance mechanisms might vary in their effectiveness against different wood decay pathogens.

### Latent infection of decay diseases in asymptomatic seedlings

Due to relatively high disease incidence rates, all seedlings inoculated by Armo, Conw, Heto, and Pors, including non-inoculated controls, were sampled for ITS-NGS analysis. Of seedlings inoculated, ITS-NGS detected pathogen-positive rates of 90% for Armo and Pors; and 80% for Conw and Heto. In contrast, all of eight non-inoculated control seedlings were asymptomatic, with the exception of one that was diagnosed as Conw positive.

For all seedlings inoculated by four pathogens, ITS-NGS detected disease infection significantly higher than the visual assessment that was based on the disease symptoms (X^2^ test, p < 0.001). In the root collar and fine root samples of asymptomatic seedlings, ITS-NGS revealed pathogen-positive rates at 60%, 75%, 83%, and 88% for inoculation by Heto, Pors, Conw, and Armo, respectively. Presence of decay pathogens in living tissues of asymptomatic seedlings indicated that latent infections occurred but had not yet caused symptoms compared to symptomatic seedlings.

**Figure 4:**
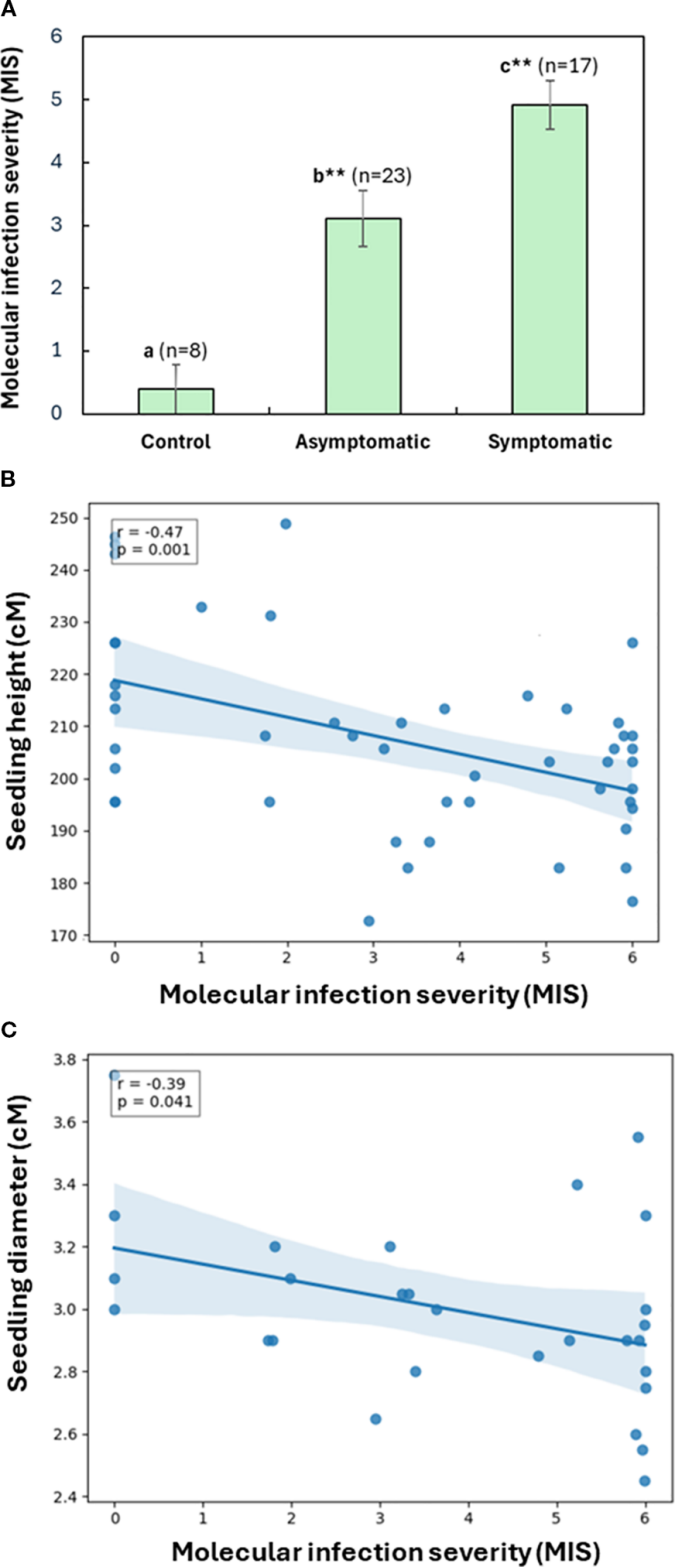
Assessment of molecular infection severity (MIS) in western redcedar seedlings based on the relative abundance of the target fungal pathogens. A total of 48 seedlings were analyzed, including 40 treatments and eight controls inoculated using both the block-stick and dowel-plus methods. **(A)** MIS comparison among symptomatic, asymptomatic, and control seedlings. The bar plot presented average MIS values with the standard error of the mean (SEM) for each seedling group. As compared to non-inoculated control, significant difference is labelled by different letters with asterisks for symptomatic and asymptomatic seedlings, respectively (one-Way ANOVA with *post-hoc* Tukey HSD test, **p < 0.01). **(B)** Relationship between seedling MIS and their heights of 48 seedlings. **(C)** Relationship between seedling MIS and their stem diameters at root collars, including 24 inoculated seedlings and four controls using the block-stick method. A linear regression shows a significant negative correlation. Shaded area represents the 95% confidence interval.

Molecular infection severity (MIS, Equation 1) showed the highest average level in symptomatic seedlings, and intermediate average level in inoculated but asymptomatic seedlings compared to non-inoculated controls (One-way ANOVA, p < 0.01; **Figure 4A**). However, among asymptomatic seedlings inoculated by four pathogens, MIS did not differ significantly between pathogens (One-way ANOVA with *post-hoc* Tukey HSD test, p > 0.05).

The GLMM test (Equation 2) revealed that the presence of fungal ITS was affected by fungal type and inoculation method (**Table 1**). Armo and Pors both showed 154 times increase in the probability of ITS presence compared to control, and Conw and Heto both showed 59 times increase the presence of fungal ITS compared to control. The dowel-plug inoculation method had only 7% of the odds of ITS presence compared to the block inoculation method, meaning that the dowel-plug inoculation method was less effective for successful infection.

**Table 1:** The effect of fungal type and inoculation type on the presence of fungal ITS (Equation 2) in redcedar.

Parameter	Estimate	Std. err.	P value	Odds ratio
^1^Fungal type:				
Armo	5.04	1.794	0.00489	154.03
Conw	4.09	1.619	0.0112	59.44
Heto	4.09	1.619	0.0112	59.44
Pors	5.04	1.79	0.0049	154.03
Inoc Dowel	-2.53	1.15	0.0272	0.079
AIC	38.29			

^1^Fungal types: Armo, *Armillaria ostoyae*; Conw, *Coniferiporia weirii*; Heto, *Heterobasidion occidentale*; Pors, *Poriella subacida*.

### Seedling growth impacted by decay diseases and latent infection

The LME model (Equation 3) revealed that height and diameter were reduced by two fungal inoculation methods (**Tables 2**, **3**) with Armo and Heto causing the most reduction of seedling heights (p = 0.01) compared to control. Conw and Poss also caused height reduction, but marginally non-significant (0.05 < p < 0.06).

**Table 2:** The effect of fungal type, inoculation method and family on height growth (cm) of redcedar (Equation 3).

Parameter	Estimate	Std. err.	P value
A) Fixed effects			
Intercept (Control)	220.1	6.03	0
^1^Fungal types:			
Armo	-21.02	7.1	**0.0103**
Conw	-15.69	7.1	0.0533
Heto	-21.1	7.1	**0.0101**
Obbr	-13.49	7.1	0.0958
Pors	-14.68	7.1	0.0703
Porp	-9.85	7.1	0.2218
Posb	-15.45	7.1	0.0572
Poss	-9.87	7.1	0.2211
Inoc Dowel	5.24	3.66	0.1559
B) Random effects		Std. dev	
Family		0.05	
Error		0.26	
AIC	710		

^1^Fungal types: Armo, *Armillaria ostoyae*; Conw, *Coniferiporia weirii*; Heto, *Heterobasidion occidentale*; Pors, *Poriella subacida*; Obbr, *Obba rivulosa*; Porp, *Porodaedalea pini*; Posb, *Postia balsamea*; Poss, *Postia sericeomollis*. Significant P values are shown in bold.

**Table 3:** The effect of fungal type, inoculation method and family on diameter growth (cm) of redcedar (Equation 3).

Parameter	Estimate	Std. err.	P value
A) Fixed effects			
Intercept (Control)	3.29	0.09	0
^1^Fungal type:			
Armo	-0.41	0.12	**0.0006**
Conw	-0.26	0.12	**0.0285**
Heto	-0.47	0.12	**0.0001**
Obbr	-0.13	0.12	0.2353
Pors	-0.22	0.12	0.063
Porp	-0.21	0.12	0.0691
Posb	-0.37	0.12	**0.0021**
Poss	-0.44	0.12	**0.0003**
Inoc Dowel	-0.15	0.05	**0.0066**
B) Random effects		Std. dev	
Family		0.01	
Error		0.24	
AIC	48.90		

^1^Fungal types: the same as listed in **Tables 1**, **2**.

Significant P values are shown in bold.

Diameters were reduced by eight pathogens, but three of them (Pors, Porp, and Obbr) were not significantly different from control for diameter growth (**Table 3**). The inoculation method only significantly affected diameter growth with the dowel-plug method producing slightly greater diameter reduction than the block-stick method (**Table 3**). No interaction was detected between fungal type and inoculation methods, indicating that the effect of fungal type was similar between inoculation methods. Family only had minor effects explaining less than 3% of the total error for height or diameter.

Replacing inoculation method with decay/discolor or pathogen ITS presence (Equation 4) indicated that only ITS had a significant effect on height growth (p = 0.03), reducing it by 7% and explaining all the effect of fungal type, indicating a general host physiological response to fungal infection, regardless of pathogen type and symptom development.

In consistency with the results above, plotting of seedling growth against MIS by a linear regression model showed that MIS was significantly negatively correlated with seedling height (r = - 0.47, N = 48, p < 0.001), regardless of inoculation method (**Figure 4B**). MIS was also negatively correlated with diameter, but only significantly in seedlings inoculated using the block-stick method (r = - 0.39, N = 28, p = 0.04, **Figure 4C**). This correlation between MIS and diameter was not significant in seedlings inoculated using the dowel-plug method (r = - 0.11, N = 20, p = 0.66), indicating that wounding from the dowel-plug method may mask the effects of fungal infection on diameter growth, while height remains a more consistent indicator of fungal impact.

## Discussion

### Controlled inoculation methods for successful infection of redcedar seedlings

This study aimed to evaluate infection rates, disease progression, and seedling growth responses following artificial inoculation with various wood decay fungal pathogens. Two inoculation methods, block-stick and dowel-plug, were compared under controlled greenhouse conditions. The block-stick method simulates natural infection by exposing living root tissues to fungal inoculum, while the dowel-plug method bypasses the epidermis and cortex, introducing the fungus directly into wood tissues via stem wounds. It is important to note that the dowel-plug method can influence susceptibility to infection in two ways. It may increase risk by compromising physical barriers, creating a moist, nutrient-rich microenvironment favorable to microbial growth. Conversely, it may decrease risk by wound-induced defenses that improve resistance to pathogens.

Despite these differences, the GLMM analysis indicated that the inoculation method did not significantly affect the incidence of disease symptoms (wood decay or discoloration). Disease incidence rates were similar between two inoculation methods across all eight tested pathogens.

However, the dowel-plug method was less effective at establishing fungal infection and had a more pronounced negative impact on stem diameter growth compared to the block-stick method. This is likely due to the greater inoculum load and longer exposure time associated with the block-stick method, which also avoids wounding damage or wound-induced defense responses. The block-stick effectiveness was further supported by extensive fungal colonization of soil and root tissues, demonstrating that these fungi can infect trees via root contact without wounding.

Root contact is a common infection pathway for root and butt rot pathogens, which spread from infected roots to healthy trees (Cleary et al., 2012; Lewis, 2013). This pathway is especially important for Conw, whose fruiting bodies are rarely observed in the field, and for which spore-based infection has not been confirmed (Sinclair and Lyon, 2005). Therefore, the block-stick method is recommended for artificial screening of redcedar resistance to root and butt rot diseases in breeding programs.

### Varying levels of redcedar resistance to different decay pathogens

Visual phenotypic assessments revealed that five decay pathogens caused wood discoloration and/or decay, with disease incidence rates ranging from 10% to 60% in five-year-old seedlings at 18 months post-inoculation. Among the eight tested pathogens, Conw and Pors were the most virulent, causing both wood decay and discoloration. To our knowledge, this finding marks the earliest onset of decay in the youngest western redcedar seedlings inoculated with these pathogens (Sturrock and Reynolds, 1998; Cleary et al., 2012). Previous trials with Conw did not observe discoloration or decay at the root collar within 12–15 months (Sturrock and Reynolds, 1998). Other pathogens, including Armo, Heto, and Posb, caused wood discoloration but did not produce decay by the end of the trial. Notably, discoloration caused by Armo and Heto began only after the fungi penetrated the vascular cambium and spread slowly upward, a pattern distinct from the more aggressive pathogens (Conw, Pors, and Posb).

Three other fungi, Porp, Poss, and Obbr, did not cause any visible symptoms or significantly affect seedling growth, suggesting that infection did not occur. This is expected for Obbr, which is primarily a saprotroph decomposing dead plant material (Miettinen and Rajchenberg, 2012). Although Porp and Poss are known to infect conifers and cause heartwood decay, they did not affect redcedar seedlings in this trial. These results indicate that redcedar exhibited variable resistance to different decay pathogens. Future resistance screening should prioritize Conw and Pors, particularly in coastal redcedar breeding programs. While this study is the first to compare redcedar responses to multiple decay pathogens under greenhouse conditions, a future field trial with larger populations is required to confirm these findings.

### Infection court and symptom development of wood decay

The root system of western redcedar is characterized by shallow, wide-spreading lateral roots and consistent development of fine roots just below the soil surface (Eis, 1974). This unique architecture allows efficient uptake of water and nutrients but also increases vulnerability to root stress caused by drought and soilborne pathogens. In this study, inoculation using the block-stick method revealed extensive colonization of fine roots by decay fungal mycelium. These fine roots lack protective bark tissue, making them more susceptible to pathogenic invasion.

ITS-NGS-based molecular diagnostics confirmed the presence of pathogens in fine roots, providing additional evidence that infection can initiate in these tissues. The observed colonization patterns suggest that infection primarily occurs in smaller roots, as very few lesions were detected at the root collar in seedlings inoculated via the block-stick method. Interestingly, one control seedling out of eight tested positive for the Conw-ITS presence. This may have resulted from natural infection via airborne spores settling into the soil, as greenhouse conditions were conducive to the development of Conw fruiting bodies.

Following initial infection, fungal mycelium invaded vascular tissues and spread proximally to the main lateral roots and root collar, causing wood discoloration and decay. Decay fungi are estimated to spread along roots at a rate of 20–40 cm per year (Sinclair and Lyon, 2005). In a controlled field inoculation of 15-year-old redcedar trees, Armo was found to have reached the vascular cambium in 42% of root samples within 12 months post-inoculation (Cleary et al., 2012).

Wood discoloration is a typical host defense response involving the production and accumulation of polyphenols, lignin, and other secondary metabolites. This study identified two distinct patterns of discoloration following successful infection by five decay fungal pathogens. Conw, Pors, and Posb caused discoloration in wood tissues of the inoculation year and/or the following year. In contrast, Armo and Heto produced more variable discoloration patterns in the root collar tissues, appearing from the cambial tissue beneath the bark and extending toward the pith. This colonization pattern likely indicates a strong defense response in cambia, contributing to the slower upward spread of these fungi. Previous microscopic examinations showed that Armo mycelium killed redcedar root cambium and induced the formation of necrophylactic periderm in the bark and callus tissue at lesion margins, effectively compartmentalizing the fungus and limiting its spread to adjacent root tissues (Cleary et al., 2012).

Callogenesis was also observed in the cambial region of main lateral roots surrounding the bases of fine roots killed by Conw in 29-year-old redcedar trees at seven years post field inoculation. However, this response did not prevent decay of the heartwood (Sturrock and Pellow, 2013). At infection sites with fine root mortality, discoloration zones were present in wood year rings formed during the first two years post-infection. In contrast, wood adjacent to the initial penetration sites showed discoloration limited to the year ring formed in the first year post inoculation. A similar pattern was observed at four-years post-inoculation in seven-year-old seedlings (Cruickshank, personal communication). Because heartwood formation in western redcedar appears to begin at a relatively small stem diameter (~7 cm) and a young age, likely between 10 and 15 years (DeBell and Lachenbruch, 2009), the discoloration zones that formed in the sapwood acted as boundaries, separating decayed inner wood from newly formed living tissues and thereby restricting horizontal fungal spread.

Taken together, evidence from multiple independent inoculation trials reveals a consistent pattern of Conw decay development in both young seedlings and mature trees. This suggests that resistance traits identified and selected in young seedlings may remain effective as trees age, supporting the potential for early-stage screening in redcedar breeding programs.

### Latent infection in wood tissues of young redcedar seedlings

Latent infection is a critical factor in evaluating quantitative resistance in tree breeding programs, particularly for long-lived species such as western redcedar. Latent infection is defined as a host without external or internal symptoms but with the presence of pathogens in sufficient quantity to cause diseases. In this study, ITS-NGS-based molecular diagnostics revealed that all four tested wood decay fungal pathogens, Armo, Conw, Pors, and Heto, were capable of establishing latent infections in redcedar seedlings. A substantial proportion of inoculated seedlings remained asymptomatic, yet fungal DNA was detected in their root collar wood tissues, confirming the presence of infection.

We found that growth responses to infection varied depending on the pathogen and the infection court. Height growth was consistently sensitive to fungal presence, regardless of symptom development or pathogen identity, suggesting a general physiological response to infection in both symptomatic and asymptomatic seedlings. In contrast, diameter growth was more significantly influenced by fungal species and inoculation method, indicating that stem development was affected by infection court and specific host–pathogen interactions. Notably, the presence of visible symptoms did not reliably correlate with growth suppression, whereas fungal quantities in tissues did. Some asymptomatic seedlings exhibited reduced growth, while certain symptomatic individuals maintained normal development. These findings imply that latent fungal activity can affect host physiology independently of symptom expression, complicating assessments based solely on visual symptoms.

The detection of latent infections poses significant challenges for breeding programs, as long-term trials are required to assess disease expression for resistance traits. Identifying asymptomatic infection stages provides critical insight into how pathogens persist within hosts, evade immune responses, and transition to active disease under favorable conditions. Future redcedar breeding efforts must account for the possibility of asymptomatic carriers, and the integration of molecular diagnostics will be essential for monitoring latent infections throughout resistance selection cycles.

Among the four pathogens studied for latent infection, Armo exhibited a low disease incidence rate (20%) and did not cause visible decay in seedlings. However, the incidence of latent infection was relatively high, albeit with lower MIS index compared to the other pathogens. This aligns with previous reports of other species documenting Armo as a contiguous fungal organism capable of persisting in asymptomatic trees for extended periods (Ferguson et al., 2003; Luana et al., 2015). Although field surveys have recorded young redcedar trees being girdled and killed by Armo (Buckland, 1946; van der Kamp, 1988), some individuals appear to possess host-mediated defense mechanisms that limit root lesion spread. In contrast, this pathogen spreads more aggressively in Douglas-fir and western hemlock (Cleary et al., 2012). Current evidence suggests that Armo and Heto exhibit lower virulence toward western redcedar compared to other forest tree species (Liu et al., 2018; Caballero et al., 2023).

Latent infections were also observed for Conw, Pors, and Heto, though the duration of their asymptomatic phases remains unknown. It is hypothesized that overt pathogenesis may follow the latent stage under environmental conditions favorable to the pathogens (Hendry et al., 2002), potentially leading to decay development or fruiting body emergence as part of the fungal life cycle. The variable incidence rates of both disease symptoms and latent infections suggest that redcedar may possess a diverse set of resistance mechanisms tailored to different pathogens. However, the molecular defense pathways and genetic architecture underlying latent persistence and transition to active disease remain largely unexplored in tree species (Parfitt et al., 2010; Vasaitis, 2013; Luana et al., 2015).

Understanding the frequency and dynamics of latent infections is essential for predicting disease outbreaks, managing forest health, and developing early intervention strategies. This study found that four decay pathogens infected a large proportion of redcedar seedlings without causing visible symptoms, emphasizing the need for nursery-level monitoring. Young seedlings may be particularly vulnerable to pathogen attack, and minimizing latent infection levels during nursery production could significantly reduce the risk of decay development after field planting. Furthermore, wound-based dowel-plug inoculation demonstrated that mechanical damage during planting may increase latent infection incidence, reinforcing the importance of careful handling practices.

Consistent with previous studies in other tree species (Luana et al., 2015; Luo et al., 2024), this research confirmed that visual assessments underestimate infection levels of decay diseases in redcedar. The prolonged latent or endophytic phase of pathogenic fungi also presents challenges for plant quarantine systems (Marsberg et al., 2017; EFSA et al., 2018), as infections often remain undetected in living seedlings until other stress triggers symptom development. Traditional nursery surveys have documented asymptomatic persistence of fungal pathogens (Stanosz et al., 2005), highlighting the limitations of symptom-based inspections. The development of molecular tools, such as ITS-NGS and quantitative PCR (qPCR), has significantly improved the sensitivity and reliability of latent infection detection in both nursery and field samples (Houston et al., 2025; Negahban et al., 2024), offering a promising path forward for early detection and pathogen containment in plant trade.

Although this study detected latent infection incidence rates ranging from 60% to 88% across the four pathogens, further validation is needed using multiple molecular diagnostic platforms, larger populations, and extended trial durations. Additionally, quantifying latent infection dynamics and monitoring reactivation events across diverse host genotypes and environmental conditions will enable more accurate assessments of redcedar vulnerability. These insights will support proactive disease management strategies, including early detection, targeted breeding for resistance, and site-specific silvicultural interventions.

## Data Availability

The datasets presented in this study can be found in the article/**Supplementary Material**.
